# The Best Age for Pregnancy and Undue Pressures 

**Published:** 2016-09

**Authors:** Carlo Bellieni

**Affiliations:** Department of Pediatrics and Maternal Health, University Hospital, Siena, Italy

**Keywords:** Motherhood, Offspring

## Abstract

In western countries we assist at the paradox that fertility is socially discouraged by a mindset that depicts fertility as a resource to exploit as late as possible. So, couples have high expectative about the advantages of delayed parenthood, but they are scarcely informed about its risks. Scientific data suggests to anticipate the first pregnancy, but social pressures impose to wait, though delayed childbearing can provoke sterility and a greater gap between generations. The best age to become parents should be autonomously decided by a couple, under the condition of being a free informed choice and not a social imposition, but currently this is not guaranteed to western women and men.

## Introduction

The U.S. fertility rate has decreased to the lowest point, according to new federal data (1). The first quarter of 2016 brought 59.8 babies for every 1,000 women, ages 15 to 44, and it is nearly half the rate at the peak of the baby boom in the late 1950s. These figures show an unmistakable trend: women who choose to reproduce keep delaying motherhood**. **In Western countries, women delay the age of the first pregnancy, in some cases well beyond their forties (2). Women’s mean age of first-time pregnancy increased in US from 21 to 25 years in the 40 years after 1970, with a decrease of mothers younger than 20 years of age, and a sensible increase of those older than 35 (3, 4). In other western countries the trend was similar, with a minimum maternal age increase of 3 years in Sweden and a maximum (4.6 years) in Denmark, while in Switzerland the mean maternal first/child age in 2006 was 29.4 years (3). In UK the average age of mothers in 2013 increased to 30.0 years, compared with 29.8 years in 2012 (5). The over-35’s now have the fastest growing birthrates and women having babies in their 40’s have more than doubled in ten years: 9,336 vs 27,000 British babies were born to women over 40 in 1989 and 2010 respectively, so that one in five British women is 35 or older when she gives birth, and deliveries to mothers aged 20 to 24 were also reduced by 8.6 per cent in 2013-14. British women aged 35 to 39 doubled their birth rate up to 64.5 conceptions per thousand women since 1990 (6). This leads to an unprecedented event: every 100 years population renews itself only 2.5 times, while 50 years ago this happened 4 times every century. This is a "honey-drop" phenomenon: time induces generations to become increasingly thinner and more widely separed, like drops of honey under the effect of the gravity.

Teen pregnancies are a risk for both women and offspring and should be discouraged; but they are a minority (7). We are assisting to a sudden increase of older pregnancies, that are likely to result in adverse outcomes (8-13). Stillbirth, miscarriage and ectopic pregnancy as well as multiple births and congenital malformations are some of these risks (14, 15). Pregnancies in women aged over 35 years are seldom associated with complications, premature births and interventions at birth and an increased risk of specific fetal abnormalities, including structural and chromosomal abnormalities (14, 16): for instance the risk of having a baby with Down syndrome exponentially increases after 36 years of maternal age. But postponing pregnancy is also a risk for women. Delayed childbearing provokes an increase of sterility among the population (17, 18). Older mothers also risk eclampsia, uterus rupture and diabetes (19). Postponing pregnancy is also a risk for the society that renews itself slowly, with social and psychological consequences that begin to be investigated. However, delaying parenthood can have beneficial aspects that are constantly highlighted by mass media: older mothers are better educated and financially stable they have the emotional maturity and life experience that translates well to motherhood (20, 21). They are more likely to breast feed and breast feed for longer (22).

Some causes of delaying parenthood

Some authors report that “the choice to delay motherhood is not so voluntary” (23). Career planning discourages maternity until a stable job is obtained. A pregnancy can interfere with a woman’s career it may affect the maternity leave allowances and new parents have difficulties in taking maternity leave because of their financial situation (24-26). Women perceive a lack of choice in the timing of when to start a family: “Women do not perceive that they have ultimate control when it comes to the timing of childbearing” (27). To overcome this situation, some companies proposed to pay for women on the payroll to freeze their eggs to permit them to delay pregnancy (28).

In industrialized countries, delayed childbearing is common for several reasons: career, financial security, fulfillment of personal goals, travel, illness; sometimes it takes years finding the ‘right partner’, sometimes divorces lead to older marriages with further children later in a second marriage. Sometimes it can take time until women feel sure they are emotionally and psychologically ready for this enormous life change (29). Finally many women spend years trying to conceive and many experience multiple miscarriages before falling pregnant. Nevertheless, in some cases the external conditions are sometimes a hard social imposition for most women, who are not always well aware of the risks of postponing a pregnancy. Delayed childbearing can also be due to scarce information or poor understanding of its connections with pregnancy complications and increased risk of adverse infant outcomes (6,30,31). This is due to the diffuse idea of omnipotence of modern medicine, or simply to disinformation, fatalism or to the trend of the moment which supposes that “thirties are the new twenties” (32).

Nevertheless, in several cases a really free choice is at the basis of delayed motherhood: women choose to postpone the first pregnancy, without any external pressure, for the personal decision that in youth they have other priorities, and in this case, women and their partners take the free responsibility of their choice. Most young women and men perceive that they have not enhanced emotional preparedness and maturity for parenting which was of benefit to both their children and themselves (33). However, the pressure done by mass media to depict pregnancy as “the icing on the cake” is evident. And this makes less free the choice of delaying pregnancy.

The best age

US data on delayed motherhood are the effect of a strong social pressure: a constant economic and social pressure to delay pregnancy without any perspective to invert this trend (1). This is a paradox. On one side scientific literature more and more clearly says that the less risky range of maternal age to bear babies is 20-30 years and on the other side, people perceive they should postpone pregnancy (31, 34). 

Thus, the best age to become parents is a compromise between two poles, and women as well as couples find it hard defending their right to having babies when young. Motherhood is represented by media as a misfortune to young people, and it is suggested to avoid it as far as possible; this is comprehensible because in past years unwanted and too young pregnancies were common, and being childless was depicted as misfortune; but nowadays the problem is the opposite: to show that a life with a baby can start even before the thirties, because, unlikely most mass media say, the thirties are not the new twenties, at least as fertility is concerned. 

Can media become more pregnancy-friendly and politics change its way of considering babies as a burden for the society, devoting to them only scarce and secondary funds and resources? To this aim, a pregnancy-friendly environment is to be created, creating the socioeconomic conditions to raise a family whenever a woman or a couple decides: a social scenario that allows only late pregnancies is unfair and politics should behave actively (e.g. with kindergartens, jobs politics and student loans) helping young people to become parents at the really desired age.

## Conflict of Interests

Authors have no conflict of interests.

## References

[1] Rossen LM, Osterman MJK, Hamilton BE, Martin JA (2016). Quarterly provisional estimates for selected birth indicators, 2014–Quarter 1, 2016 National Center for Health Statistics  National Vital Statistics System, Vital Statistics Rapid Release Program.

[2] Carter D, Watt AM, Braunack-Mayer A, Elshaug AG, Moss JR, Hiller JE (2013). Should there be a female age limit on public funding for assisted reproductive technology?. J Bioeth Inq.

[3] Matthews TJ, Hamilton BE (2009). Delayed childbearing: more women are having their First child later in life. NCHS Data Brief.

[4] Martin JA, Hamilton BE, Ostermann MJK (2013). Births: Final data for 2012. Natl Vital Stat Rep.

[5] Briggs H (2014). ONS: Mothers' average age hits 30.

[6] Parry L (2015). Average age of UK mother is 34 as number of babies born falls to seven-year low. Dailymail.

[7] Goisis A, Sigle-Rushton W (2014). Childbearing postponement and child well-being: a complex and varied relationship?. Demography.

[8] Balasch J, Gratacós E (2012). Delayed childbearing: effects on fertility and the outcome of pregnancy. Curr Opin Obstet Gynecol.

[9] Mazza D, Cannold L, Nagle C, McKay F, Brijnath B (2012). Making decisions about fertility--three facts GPs need to communicate to women. Aust Fam Physician.

[10] Bellieni CV (2012). Neonatal risks from in vitro fertilization and delayed motherhood. World J Clin Pediatr.

[11] Alonzo AA (2002). Long-term health consequences of delayed childbirth: NHANES III. Womens Health Issues.

[12] Breart G (1997). Delayed childbearing. Eur J Obstet Gynecol Reprod Biol.

[13] Andersen A-MN, Wohlfahrt J, Christens P, Olsen J, Melbye M (2000). Maternal age and fetal loss: population based register linkage study. BMJ.

[14] Baird DT, Collins J, Egozcue J, Evers LH, Gianaroli L, Leridon H (2005). Fertility and ageing. Hum Reprod Update.

[15] Stein Z, Susser M (2000). The risks of having children in later life. West J Med.

[16] Kovacs P (2010). The down side of delayed childbearing.

[17] Spandorfer SD, Bendikson K, Dragisic K Schattman G, Davis OK, Rosenwaks Z (2007). Outcome of in vitro fertilization in women 45 years and older who use autologous oocytes. Fertil Steril.

[18] Nyboe Andersen A, Goossens V, Bhattacharya S, Ferraretti AP, Kupka MS, de Mouzon J (2009). Assisted reproductive technology and intrauterine inseminations in Europe, 2005: results generated from European registers by ESHRE: ESHRE The European IVF Monitoring Programme (EIM), for the European Society of Human Reproduction and Embryology (ESHRE). Hum Reprod.

[19] Salihu HM, Shumpert MN, Slay M, Kirby RS, Alexander GR (2003). Childbearing beyond maternal age 50 and fetal outcomes in the United States. Obstet Gynecol.

[20] Sonobe M, Usui M, Hiroi K, Asai H, Hiramatsu M, Nekoda Y (2016). Influence of older primiparity on childbirth, parenting stress, and mother-child interaction. Jpn J Nurs Sci.

[21] Lampinen R, Vehviläinen-Julkunen K, Kankkunen P (2009). A review of pregnancy in women over 35 years of age. Open Nurs J.

[22] Grimshaw KE, Aksoy B, Palmer A, Jenner K, Oliver EM, Maskell J (2015). Prospective food diaries demonstrate breastfeeding characteristics in a UK birth cohort. Matern Child Nutr.

[23] Petropanagos A (2010). Reproductive 'choice' and egg freezing. Cancer Treat Res.

[24] Bianchi SM (2011). Changing families, changing workplaces. Future Child.

[25] Zhang X (2009). Earnings of women with and without children. Perspectives. Statistics Canada Ed.

[26] Beaupré P, Cloutier E (2007). Navigating Family Transitions: Evidence from the General Social Survey, 2006. General Social Survey, Cycle 20.

[27] Cooke A, Mills TA, Lavender T (2012). Advanced maternal age: Delayed childbearing is rarely a conscious choice A qualitative study of women's views and experiences. Int J Nurs Stud.

[28] Friedmann D (2014). Perk Up: Facebook and Apple Now Pay for Women to Freeze Eggs.

[29] Benzies K, Tough S, Tofflemire K, Frick C, Faber A, Newburn-Cook C (2006). Factors influencing women's decisions about timing of motherhood. J Obstet Gynecol Neonatal Nurs.

[30] Tough S, Tofflemire K, Benzies K, Fraser-Lee N, Newburn-Cook C (2007). Factors influencing childbearing decisions and knowledge of perinatal risks among Canadian men and women. Matern Child Health J.

[31] Gossett DR, Nayak S, Bhatt S, Bailey SC (2013). What do healthy women know about the consequences of delayed childbearing?. J Health Commun.

[32] Jay M (2012). The Defining Decade: Why Your Twenties Matter--And How to Make the Most of Them Now.

[33] MacDougall K, Beyene Y, Nachtigall RD (2012). 'Inconvenient biology:' advantages and disadvantages of first-time parenting after age 40 using in vitro fertilization. Hum Reprod.

[34] American College of Obstetricians and Gynecologists Committee on Gynecologic Practice and Practice Committee (2014). Female age-related fertility decline. Committee Opinion No. 589. Fertil Steril.

